# Supplementary material for: The landscape of hereditary haemochromatosis risk and diagnosis across the British Isles and Ireland
Source: Nat Commun. 2026 Feb 3;17:716. doi: 10.1038/s41467-025-65511-7 (PMC12868708; doi:10.1038/s41467-025-65511-7)

Supplementary Figure 1: Map of expected frequencies of the secondary risk genotype, HFE p.Cys282Tyr/p.His63Asp compound heterozygotes.

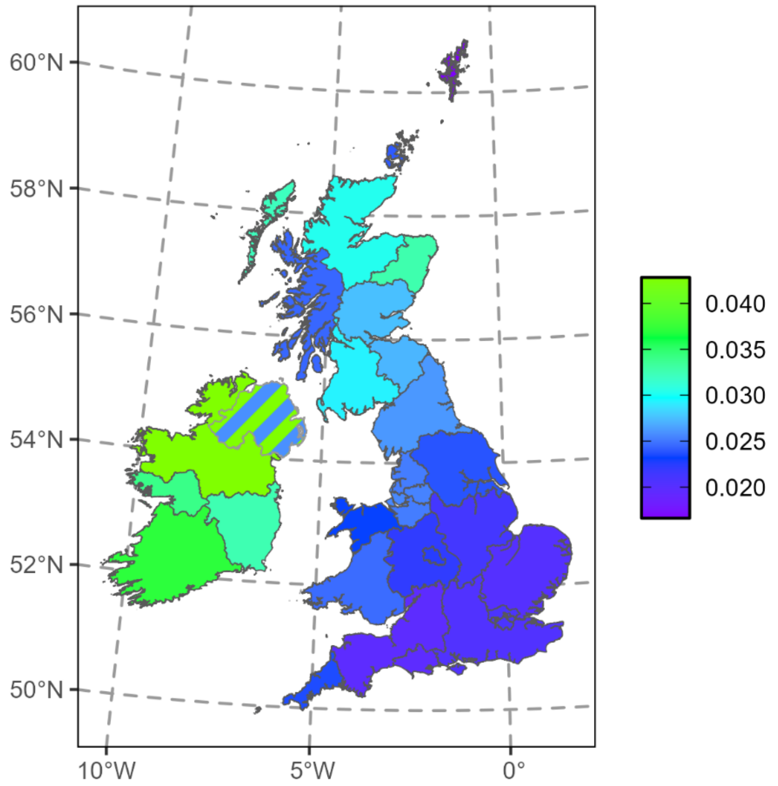

Supplementary Figure 2: Map of overall genetic risk arising from the two major risk variants in HFE. The predicted genotype frequencies are weighted by their penetrance, and plotted for males.

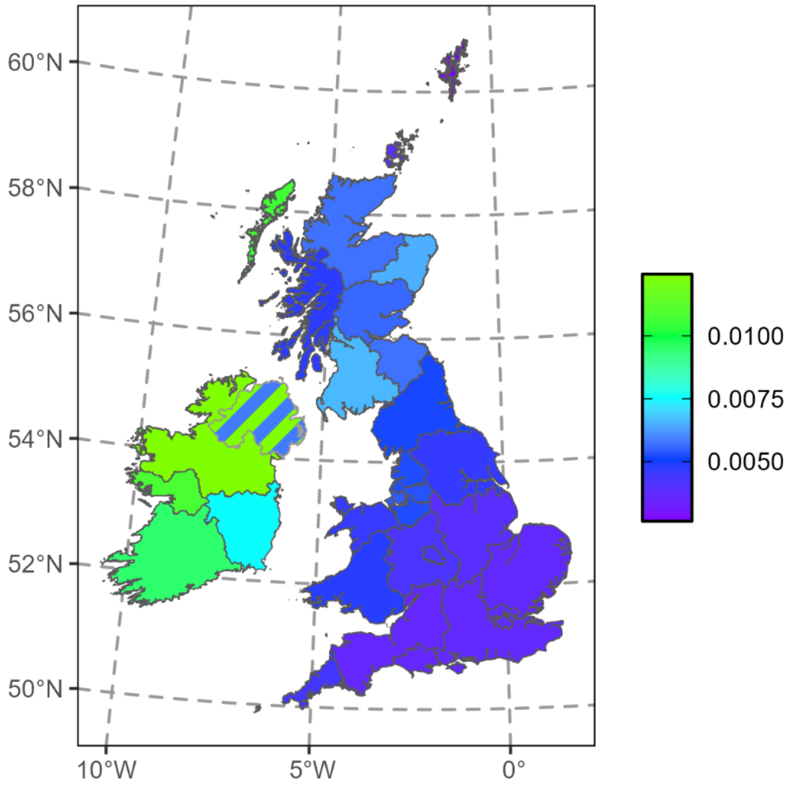

Supplement: Supplementary file 1 — Supplementary Information [file 41467_2025_65511_MOESM1_ESM.pdf]
