# Supplementary material for: The landscape of hereditary haemochromatosis risk and diagnosis across the British Isles and Ireland
Source: Nat Commun. 2026 Feb 3;17:716. doi: 10.1038/s41467-025-65511-7 (PMC12868708; doi:10.1038/s41467-025-65511-7)
Supplement: Supplementary file 8 — Reporting Summary [file 41467_2025_65511_MOESM8_ESM.pdf]

nature portfolio

Corresponding author(s): James F Wilson

Last updated by author(s): Feb 19, 2025

Reporting Summary

Nature Portfolio wishes to improve the reproducibility of the work that we publish. This form provides structure for consistency and transparency in reporting. For further information on Nature Portfolio policies, see our Editorial Policies and the Editorial Policy Checklist.

Statistics

For all statistical analyses, confirm that the following items are present in the figure legend, table legend, main text, or Methods section.

n/a	Confirmed	
☐	☒	The exact sample size (*n*) for each experimental group/condition, given as a discrete number and unit of measurement
☒	☐	A statement on whether measurements were taken from distinct samples or whether the same sample was measured repeatedly
☐	☒	The statistical test(s) used AND whether they are one- or two-sided<br>*Only common tests should be described solely by name; describe more complex techniques in the Methods section.*
☒	☐	A description of all covariates tested
☒	☐	A description of any assumptions or corrections, such as tests of normality and adjustment for multiple comparisons
☐	☒	A full description of the statistical parameters including central tendency (e.g. means) or other basic estimates (e.g. regression coefficient) AND variation (e.g. standard deviation) or associated estimates of uncertainty (e.g. confidence intervals)
☐	☒	For null hypothesis testing, the test statistic (e.g. F, t, r) with confidence intervals, effect sizes, degrees of freedom and P value noted<br>*Give P values as exact values whenever suitable.*
☒	☐	For Bayesian analysis, information on the choice of priors and Markov chain Monte Carlo settings
☒	☐	For hierarchical and complex designs, identification of the appropriate level for tests and full reporting of outcomes
☒	☐	Estimates of effect sizes (e.g. Cohen's *d*, Pearson's *r*), indicating how they were calculated

Our web collection on statistics for biologists contains articles on many of the points above.

Software and code

Policy information about availability of computer code

Data collection

No software was used for data collection.

Data analysis

All software used in this work is publicly available. It is described (with versions) throughout the main text of the manuscript and repeated here for convenience:
R version 4.4.2
R package dbscan v1.2-0
R package ggplot2 v3.5.1
KING v2.3.2
EAGLE 2.4.1
hap-ibd v 14/06/2023
leidenalg v 0.10.2
PLINK v1.90b and v2
PRIMUS v1.9.0
NHS DigiTrials Feasibility Self-Service 31/10/2024 data freeze

All bespoke code has been deposited in GitHub (https://github.com/viking-genes/uk-hfe).

For manuscripts utilizing custom algorithms or software that are central to the research but not yet described in published literature, software must be made available to editors and reviewers. We strongly encourage code deposition in a community repository (e.g. GitHub). See the Nature Portfolio guidelines for submitting code & software for further information.

Data

Policy information about availability of data

All manuscripts must include a data availability statement. This statement should provide the following information, where applicable:

- Accession codes, unique identifiers, or web links for publicly available datasets
- A description of any restrictions on data availability
- For clinical datasets or third party data, please ensure that the statement adheres to our policy

Data Availability Statement. There is neither Research Ethics Committee approval, nor consent from participants, to permit open release of the individual level research data underlying this study. Instead, the research data are available through managed access after application to each population cohort. For UK Biobank, www.ukbiobank.ac.uk/register-apply; for Viking Genes, https://viking.ed.ac.uk/our-data-and-samples/access; in accord with the consent given by participants and favourable opinions from a Research Ethics Committee. Each DAC-approved project is subject to a data or materials transfer agreement (D/MTA) or commercial contract. Data may then be shared with academic or commercial recipients worldwide and may be used within the parameters of the study Protocols. New sequencing data were generated as part of the study. Data from routinely collected healthcare records on patients across NHS England national datasets is available on application to the NHS DigiTrials Feasibility Self-Service (https://digital.nhs.uk/services/nhs-digitrials/feasibility-service). Access to the UK Biobank genotype data was approved under applications 19655 and 103770.

Research involving human participants, their data, or biological material

Policy information about studies with human participants or human data. See also policy information about sex, gender (identity/presentation), and sexual orientation and race, ethnicity and racism.

Reporting on sex and gender	The HFE alleles we describe are autosomal and therefore assort independently of the sex chromosomes. There is no expectation of differences in allele frequencies between males and females; moreover, as dividing the samples in two would decrease the power of the study and precision of the estimates, no sex-based analyses were performed.
Reporting on race, ethnicity, or other socially relevant groupings	In Viking Genes, participants were included in the study if they had all four grandparents born in Orkney or all four in Shetland, hence there are no ethnic minority participants. From these sets, maximally unrelated subsets were selected for further analysis. In UK Biobank, we selected only participants who self-identify as "White British", exhibit very similar genetic ancestry based on a principal components analysis of the UKB genome-wide SNP array genotypes (UKB field: 22006) and who were born outside large metropolitan areas in the corresponding region. The Irish participants had to self-identify as "Irish" (UKB field: 21000) and be born in either Northern Ireland or the Republic of Ireland (UKB field: 1647). The participants satisfying the above criteria for each region were then evaluated for relatedness and the maximum unrelated set per region generated as for the Northern Isles cohorts. Ethnicity data in NHS DigiTrials is self-reported using the 2001 UK census classification.
Population characteristics	The populations analysed were not chosen on the basis of age or any phenotypic characteristic or diagnosis. They were rather unrelated healthy adult volunteer members of the respective cohorts, representing different geographic ancestries across the British Isles and Ireland. All were subject to whole exome sequencing and sub-populations were chosen according to the birthplaces of participants (for UK Biobank) or grandparents of participants (for Viking Genes). The population characteristics of the subjects will reflect those of the overall cohorts from which they are drawn.
Recruitment	UK Biobank recruitment is detailed in Allen et al 2024. Sci Transl Med doi: 10.1126/scitranslmed.adf4428. Recruitment in Viking Genes is described in Kerr et al (2019) Scientific Reports 9:10964. There is evidence of a "healthy volunteer" selection bias in the UKB (Fry et al Am J Epidemiol. 2017 Nov 1;186(9):1026-1034. doi: 10.1093/aje/kwx246), but we consider it unlikely that these biases operated to different degrees in different parts of the UK, such that they would substantively affect our conclusions
Ethics oversight	All participants in the Viking Health Study—Shetland (VIKING) gave written informed consent for broad ranging health and ancestry research including, whole genome/exome sequencing and the study was given a favourable opinion by the South East Scotland Research Ethics Committee (REC Ref 12/SS/0151). All participants in the Orkney Complex Disease Study (ORCADES) gave written informed consent for broad ranging health and population research, including sequencing and the study was approved by Research Ethics Committees in Orkney, Aberdeen (North of Scotland REC), and South East Scotland REC, NHS Lothian (reference: 12/SS/0151). All Viking Genes participants are now unified under South East Scotland REC reference: 19/SS/0104. Ethics approval for the UK Biobank study was obtained from the North West Centre for Research Ethics Committee (11/NW/0382), and all participants gave written informed consent.

Note that full information on the approval of the study protocol must also be provided in the manuscript.

Field-specific reporting

Please select the one below that is the best fit for your research. If you are not sure, read the appropriate sections before making your selection.

☒ Life sciences ☐ Behavioural & social sciences ☐ Ecological, evolutionary & environmental sciences

For a reference copy of the document with all sections, see nature.com/documents/nr-reporting-summary-flat.pdf

Life sciences study design

All studies must disclose on these points even when the disclosure is negative.

Sample size	No power calculation was performed. In population genetics the larger the sample size, the greater the ability to investigate rarer alleles, for there are more observations of each allele. The sample sizes here were constrained by the number of individuals born in each region, and we did not consider any regions with sample sizes less than ~500. The overall sample size was ~400,000.
Data exclusions	Individuals from Viking Genes who did not have all four grandparents born in either Orkney, Shetland or the Western Isles were excluded. After assessing and reporting the genotype frequencies in the major ethnic minorities and immigrant groups in the UK Biobank, we considered only the predefined white British or white Irish for the analysis across the 29 regions.
Replication	Without the recruitment of new cohorts with tens of thousands of individuals born across the 29 regions of the British Isles and Ireland, it is not possible to replicate our findings. Such a dataset does not exist at present. We note that the patterns we observe reflect the broad brush information that was known previously and don't contradict any previous publications.
Randomization	The present study does not describe a randomised controlled trial, thus allocation and randomisation are not relevant. It is rather an observational study.
Blinding	The present study does not describe a blinded randomised controlled trial, thus it was not necessary to blind researchers. Subjects analysed in this study were volunteers from the general population. Allocation, treatment and randomisation are not relevant to this study.

Reporting for specific materials, systems and methods

We require information from authors about some types of materials, experimental systems and methods used in many studies. Here, indicate whether each material, system or method listed is relevant to your study. If you are not sure if a list item applies to your research, read the appropriate section before selecting a response.

Materials & experimental systems

n/a	Involved in the study	
☒	☐	Antibodies
☒	☐	Eukaryotic cell lines
☒	☐	Palaeontology and archaeology
☒	☐	Animals and other organisms
☒	☐	Clinical data
☒	☐	Dual use research of concern
☒	☐	Plants

Methods

n/a	Involved in the study	
☒	☐	ChIP-seq
☒	☐	Flow cytometry
☒	☐	MRI-based neuroimaging

Plants

Seed stocks	n/a
Novel plant genotypes	n/a
Authentication	n/a
